# A 3D engineered multiple myeloma niche for evaluating CAR T cell therapy

**DOI:** 10.3389/fbioe.2026.1860416

**Published:** 2026-09-09

**Authors:** Lorea Jordana-Urriza, Manuele G. Muraro, Marc Du, Victoria Lambrecht, Juan Roberto Rodriguez-Madoz, Ivan Martin, Andrés García-García

**Affiliations:** 1 Hemato-Oncology Program. Cima Universidad de Navarra. IdiSNA, Pamplona, Spain; 2 Tissue Engineering, Department of Biomedicine, University of Basel and University Hospital Basel, Basel, Switzerland; 3 Centro de Investigacion Biomedica en Red de Cancer (CIBERONC), Madrid, Spain; 4 Cancer Center Clinica Universidad de Navarra (CCUN), Pamplona, Spain; 5 Tissue Engineering, Department of Biomedical Engineering, University of Basel, Basel, Switzerland

**Keywords:** 3D osteogenic niche, bone marrow, CAR T cell, immunotherapy, multiple myeloma

## Abstract

Chimeric antigen receptor (CAR) T cell therapies targeting B cell maturation antigen (BCMA) have shown unprecedented success in treating multiple myeloma (MM), yet long-term relapse remains a critical challenge, possible due to the protective influence of the bone marrow (BM) microenvironment. Current *in vitro* models often rely on simplistic 2D cocultures of CAR T and MM cells, and fail to recapitulate the 3D structural and cellular complexity of the osteogenic niche, which is known to facilitate immune evasion. In this study, we aimed to develop a physiologically relevant 3D *in vitro* MM model using a perfusion bioreactor system, hydroxyapatite scaffolds, and osteogenically-differentiated human BM mesenchymal stromal cells. Our results demonstrate that this system effectively supports the engraftment and 3D clustering of MM cells while successfully mimicking the suppressive effect of the tumor on osteogenic matrix deposition. Use of the platform to assess BCMA-targeted CAR T cell activity indicated that, while CAR T cells efficiently cleared circulating tumor cells in the fluidic phase, the 3D osteogenic niche provided a protective environment that blunted cytotoxic efficacy. This resistance was not associated with T cell recruitment, suggesting that the 3D niche provides complex protection. Our study provides a proof-of-concept for a humanized, dynamic cell culture platform capturing the influence of the BM niche-MM cell crosstalk on the efficacy of immunotherapy. The model represents a promising tool for evaluating next-generation CAR T cell potencies and for investigating associated resistance mechanisms.

## 1 Introduction

Chimeric antigen receptor (CAR) T cell therapies have become a promising therapeutic option for patients suffering from B-cell hematological malignancies, including multiple myeloma (MM) (Zhang et al., 2022). In MM, CAR T therapies targeting B-cell maturation antigen (BCMA) have produced unprecedent rates of remission and increased life expectancy for relapsed/refractory (R/R) patients. Nevertheless, almost every patient eventually relapses in the long term (Berdeja et al., 2021; Munshi et al., 2021; Rodriguez-Otero et al., 2023; San-Miguel et al., 2023). This lack of long-term response is one of the main challenges in CAR T therapies for MM and depends on multiple factors, including CAR T cell expansion and persistence capability, CAR T cell dysfunction within the tumor niche, as well as tumor escape mechanisms (Turtle et al., 2016; Garfall et al., 2019; Shah and Fry, 2019; Boulch et al., 2021; Da Vià et al., 2021; Samur et al., 2021; Li et al., 2022; Monfrini et al., 2022; Lee et al., 2023).

MM is characterized by the accumulation of malignant plasma cells within the bone marrow. This tumor microenvironment harbors a complex network of aberrant mesenchymal and hematopoietic cells that alters bone remodeling and supports malignant cells through various mechanisms, including the secretion of IL-6 and growth factors that nurture tumor proliferation (Arnulf et al., 2007; Corre et al., 2007; Garayoa et al., 2009; Wang et al., 2014; Malard et al., 2024). Osteolineage cells are not only an important source of IL-6, but also central to this pathological crosstalk, modulating tumor growth and niche remodeling through direct contact and paracrine signaling (Karadag et al., 2000; Zheng et al., 2014). The MM niche also contributes to resistance to CAR T cell therapy by limiting CAR T cell infiltration and function through physical barriers and immunosuppressive signaling (Jakubikova et al., 2016; García-Ortiz et al., 2021; Mirvis and Benjamin, 2024). Understanding how the 3D structural environment and the stroma influence CAR T cell infiltration, activation and cytotoxicity is critical for the optimization of the next-generation of CAR T cell immunotherapies. However, most *in vitro* approaches to assess CAR T cell therapies for MM often fail to recapitulate this biological complexity, consisting mostly of 2D cocultures of tumor and CAR T cells to evaluate direct killing capacity. Although several 3D MM models have been developed to study bone–tumor interactions or therapeutic responses, including CAR T cell therapy (de la Puente et al., 2015; Ilic et al., 2024; Luanpitpong et al., 2025), existing systems either lack an osteogenic compartment or do not evaluate CAR T cell function within the engineered niche (Reagan et al., 2014; Zhang et al., 2014; Jakubikova et al., 2016; Braham et al., 2018; Visconti et al., 2020; Lourenço et al., 2022).

We previously described a 3D *in vitro* model of the bone marrow (BM) niche, based on ceramic porous scaffolds and BM mesenchymal stromal cells (BM-MSCs) osteogenically differentiated under perfusion flow (Bourgine et al., 2018). Here, we further developed the model towards a pathologic setting by introducing MM cells. We first demonstrated that the engineered niche supports MM cell engraftment while allowing CAR T cell recruitment and persistence. Finally, we provided proof-of-concept that this platform enables evaluation of CAR T cell efficacy in a human-relevant 3D microenvironment that captures key aspects of niche-mediated tumor protection.

## 2 Materials and methods

### 2.1 Human primary cells

Human BM aspirates were collected from healthy donors at the University Hospital Basel. Informed consent was obtained preoperatively and the local ethics committee (Ethikkommission Nordwest-und Zentralschweiz, ref. 78/07) approved the protocol. Peripheral blood mononuclear cells (PBMCs) were isolated from buffy coats from healthy donors at the Blutspendezentrum Basel. All experiments were compliant with EU recommendations.

### 2.2 Cell lines

MM.1S-GFPLuc cell line (ATCC) was cultured in Roswell Park Memorial Institute medium (RPMI) 1640 (Gibco) supplemented with 20% fetal bovine serum (FBS) (Gibco). HEK293T (ATCC) cells were cultured in Dulbecco’s Modified Eagle Medium (DMEM) (Gibco) supplemented with 10% FBS. All media were supplemented with 1% penicillin/streptomycin (Gibco) and 1% L-Glutamine (Gibco). Cell lines were maintained at 37 °C in a humidified atmosphere with 5% CO_2_.

### 2.3 BM-MSC extraction and culture

BM aspirates (20 mL volumes) were harvested from healthy donors (n ≥ 3) using a BM biopsy needle inserted through the cortical bone and immediately transferred into plastic tubes containing 15,000 IU heparin. After diluting the marrow aspirates with phosphate buffered saline (PBS) at a ratio of 1:4, nucleated cells were isolated using Histopaque density gradient solution (Sigma; cat# 10771). Complete medium (CM) consisting of α-minimum essential Medium (αMEM) (Gibco) with 10% fetal bovine serum (FBS), 1% HEPES (1 M), 1% sodium pyruvate (100 mM) and 1% Penicillin–Streptomycin–Glutamine (PSG; 100X) solution (all from Gibco). Nucleated cells were plated at a density of 3 × 10^6^ cells/cm^2^ in CM supplemented with 5 ng/mL of fibroblast growth factor-2 (FGF-2, R&D Systems) and cultured at 37 °C in a water-jacketed incubator with 5% CO_2_. Medium was changed twice a week. BM-MSCs were selected based on adhesion and proliferation on the plastic substrate 1 week after seeding.

### 2.4 Engineering of 3D osteogenic niches in perfusion bioreactors

3D niches were generated using BM-MSCs as previously described (Bourgine et al., 2018). Briefly, hydroxyapatite scaffolds with trabecular structure and 90% porosity (Engipore®, Finceramica-Faenza, Faenza, Italy) of 4 mm height and 8 mm diameter were seeded in perfusion bioreactor with 0.75 × 10^6^ of BM-MSC at a superficial velocity of 3000 μL/min. After 24 h (cell seeding phase), the superficial velocity was reduced to 10 μL/min for the perfusion culture of BM-MSC, which was maintained over the experiment. Cells were then cultured for 1 week in proliferative medium (PM), consisting of CM supplemented with 10 nM dexamethasone (Sigma; cat# D4902), 0.1 mM ascorbic acid-2-phosphate (Sigma; cat# A92902) and 5 ng/mL FGF-2, followed by two or 3 weeks of Osteogenic Medium (OM) consisting of CM supplemented with 10 nM dexamethasone, 10 mM β-glycerophosphate, and 0.1 mM ascorbic acid-2-phosphate. Culture medium was changed twice a week.

### 2.5 Lentiviral vector construction and virus preparation

A third-generation self-inactivating LV vector (pCCL) was used to express under the EF1α promoter a second-generation CAR constructs targeting human BCMA. CAR structure comprised the scFv, a CD8 hinge and transmembrane domain, the 4-1BB co-stimulatory domain and CD3ζ endodomain fused via T2A to a truncated version of the EGFR reporter gene. Replication-defective LV vectors were produced in HEK293T cells following standard procedures. Briefly, 6 × 10^6^ HEK293T cells were plated in a p100 culture dish and after 24 h they were cotransfected with 6.9 µg LV vector, 3.41 µg pMDLg/pRRE (Gag/Pol), 1.7 µg pRSVRev and 2 µg pMD2.G (VSVG envelope) packaging plasmid using Lipofectamine 2000 (Invitrogen). Medium was changed after 6h, and after 40 h supernatants were collected and filtered with low retention 0.45 µm filters. Virus was concentrated using Lenti-X Concentrator (Takara) following manufacturer’s instructions and stored at −80 °C for future use.

### 2.6 CAR T cell generation

PBMCs were isolated from buffy coats of healthy donors using Ficoll-PaqueTM Plus (GE Healthcare). CD4^+^ and CD8^+^ cells were isolated using CD4 and CD8 MicroBeads (Miltenyi Biotec) in the QuadroMACS™ separator with LS columns (Miltenyi Biotec). Isolated T cells were cultured in 24-well plates at a concentration of 1 × 10^6^ cells/ml (at a volume of 2 mL/well) in RPMI medium 3% human serum (HS, Sigma-Aldrich) and 1% penicillin/streptomycin, supplemented with 625 IU/mL of human IL-7 and 85 IU/mL of human IL-15 (Miltenyi Biotec) (complete T cell medium); and activated with 10 μL/ml T cell TransAct (Miltenyi Biotec). After 48 h, T cells were infected with the CAR LV vector at multiplicity of infection (MOI) 4 and 10 μL/mL of LentiBoost (Sirion Biotech) to enhance viral infection. 72 h after infection CAR T cells were centrifuged, washed, counted, and adjusted to 1 × 10^6^ cells/ml. CAR T cells were counted every other day during the culture period, and the concentration was adjusted to 1 × 10^6^ cells/ml until day 12 of the production. CAR T cells were maintained at 37 °C in a humidified atmosphere with 5% CO_2_.

### 2.7 *In vitro* evaluation of CAR T cell cytotoxicity

For evaluating specific CAR T cell killing, CAR T cells were co-cultured with fresh MM.1S-GFPLuc targets in complete T cell medium starting effector-to-target (E:T) ratio of 1:1, followed by 1:3 serial dilutions in triplicate. After 24 h, specific lysis was quantified using the Bright-Glo^TM^ Luciferase Assay System (Promega), following manufacturer’s instructions. Luminescence was measured using a luminometer and normalized to spontaneous and maximum lysis controls.

### 2.8 *In vivo* evaluation of CAR T cell cytotoxicity

All experimental procedures were approved by the Ethics Committee of the University of Navarra and the Institute of Public Health of Navarra according to European Council Guidelines. NOD-SCID-IL2rg^−/−^ (NSG) mice were purchased from The Jackson Laboratory (JAX) and bred and maintained in-house in a pathogen-free facility. Eight-to-twelve-week-old male or female mice were administered 3 × 10^6^ MM.1S-GFPLuc cells intravenously through the lateral tail vein. Mice were randomized to ensure equal pre-treatment tumor burden before CAR T cell treatment. At day 14, mice received intravenous injection of 0.5 × 10^6^ BCMA CAR T cells. Physiological saline solution was used for cell resuspension in 200 μL/mice and the same volume without cells for control group. Mice were humanely euthanized when mice demonstrated signs of morbidity and/or hindlimb paralysis.

### 2.9 MM and CAR T cells seeding and culture in 3D osteogenic niches

GFP^+^MM.1S cells were seeded onto 3D niches or hydroxyapatite scaffolds (Engipore®, Finceramica-Faenza, Faenza, Italy) by overnight perfusion at superficial velocity of 3000 μL/min. Different MM.1S cell numbers were cultured: 0.1 million, 0.5 million or one million MM cells resuspended in RPMI 1640 medium (Gibco), supplemented with 20% Fetal Bovine Serum (FBS, Gibco) (MM medium). After seeding, MM.1S cells were cultured with a superficial velocity of 10 μL/min for 1 week in MM medium. The medium was changed twice weekly, and the collected medium was centrifuged (300 g, 10 min) to recover cells present in the supernatant. The pelleted cells were resuspended in fresh MM medium and injected back in the corresponding bioreactor. CAR T cells were seeded 1 week after MM.1S seeding, with a density of 1x10^6^ CAR positive (CAR^+^) T cells per bioreactor. CAR T cells were resuspended in a new medium containing RPMI supplemented with 3% HS (Sigma-Aldrich).

### 2.10 Cell harvesting from supernatants in perfusion bioreactors

Floating and loosely attached cells were collected from the medium contained in the bioreactor tubes and chamber at the end of culture period. Bioreactors were then washed with 6 mL of PBS under perfusion. Harvested cells were spun down (500 g, 5 min) and resuspended in PBS.

### 2.11 Flow cytometry

Phenotypic characterization of donor-derived T cells and CAR T cells was performed at days 0 and 14 of the production. Similarly, the immunophenotype of CAR T and tumor cells retrieved from bioreactors was also determined using LSRFortessa™ (BD Biosciences) cytometer and Flowjo 10.10 software (TreeStar). Donor-derived T cells and cells harvested from the supernatants were labelled using the following fluorescent antibody conjugates with appropriate dilution (2–5 μg/mL) in PBS 2% FBS 0.5M EDTA: human anti-CCR7 (Biolegend; cat#353204), human anti-CD45RA (Biolegend; cat#304121), human anti-CD3 (Biolegend; cat#317333), human anti-EGFR (Biolegend; cat#352911) and human anti-CD8 (Biolegend; cat#301047). Cell viability was assessed using Live/Dead staining (BD Biosciences; cat#565388). MM.1S cells were detected via GFP expression. T cell subpopulations were defined as stem cell memory/naïve, TSCM/TNAIVE (CCR7^+^, CD45RA^+^); central memory, TCM (CCR7^+^, CD45RA^−^); effector memory, TEM (CCR7^-^, CD45RA^−^); and terminal effector, TEMRA (CCR7^-^, CD45RA^+^).

### 2.12 Histological analyses

Scaffolds were encapsulated in HistoGel (Epredia™ HG-4000–012) matrix to provide mechanical support during subsequent processing. Following solidification, the HistoGel-embedded scaffolds were carefully extracted and placed into 50 mL tubes containing 40 mL PBS 15% EDTA solution for decalcification. Decalcification was performed at 37 °C under continuous orbital agitation at 70 rpm during 5–7 days until the process was complete. After decalcification, samples were embedded in paraffin and histological sections were obtained using a Microtome for hematoxylin/eosin (H&E) and immunofluorescence staining.

### 2.13 Immunostainings

Immunofluorescence staining of engineered niches was performed in histological sections and in whole mount. For histological analysis, tissue sections were first deparaffinized and rehydrated through two sequential immersions in Ultraclear (15 min each), followed by a graded ethanol series (100% for 5 min; 96%, 70%, and 35% for 1 min each), and final washes in PBS and deionized (DI) water for 5 min each. Antigen retrieval was performed by incubating the slides in a 1x citrate buffer (pH 6.0) at 96 °C for 15 min. Following incubation, the sections were cooled to 70 °C and washed in distilled water. Sections were blocked for non-specific binding using 5% bovine serum albumin (BSA) in 0.2% PBS-Tween (PBS-T) for 30 min at RT in a hydration chamber. Samples were incubated with primary antibodies diluted in 1% BSA 0.2% PBS-T at 4 °C overnight. After incubation, slides were washed twice in 1% BSA 0.2% PBS-T. Secondary antibody incubation (1:300 in 1% BSA 0.2% PBS-T) was carried out for 1 h at RT in the dark. Nuclei were counterstained with DAPI (1:1000 in PBS) for 10 min at RT. Finally, sections were mounted using Fluoromount Aqueous Mounting Medium and cover slipped. For whole mount imaging, intact 3D niches harvested from the bioreactors were fixed by incubation in 2% paraformaldehyde (vol/vol) for 24 h. After rinsing in PBS, samples were blocked and permeabilized with 3% BSA in 0.2% PBS-T for 6 h at room temperature (RT). Then, samples were washed with PBS and incubated with primary antibodies diluted in 0.5% BSA in 0.2% PBS-T at 4 °C for 2 days. The following primary antibodies were used: human anti-osteocalcin (Abcam; cat#ab93876), human anti-CD3 (Abcam; cat#ab16669). Tissues were washed with PBS 8 times and incubated with matched secondary antibodies diluted in PBS 2% BSA overnight at 4 °C. Finally, samples were washed with PBS for 4 h and counterstained for 10 min with 5 μM DAPI. Samples were stored in PBS at 4 °C until analysis. Images were acquired using a laser scanning confocal microscope (Zeiss LSM 710) and analyzed using ImageJ and QuPath softwares. For all stainings, antibody control samples were included and processed simultaneously.

### 2.14 Luminex assays

Protein content in supernatant fractions harvested from the perfusion bioreactors was determined using Human XL Cytokine Luminex Kit Performance Assay (Bio-techne; cat#FCSTM18B-10) according to manufacturer’s instructions.

## 3 Results

### 3.1 Engineering of a 3D MM model

**FIGURE 1:**
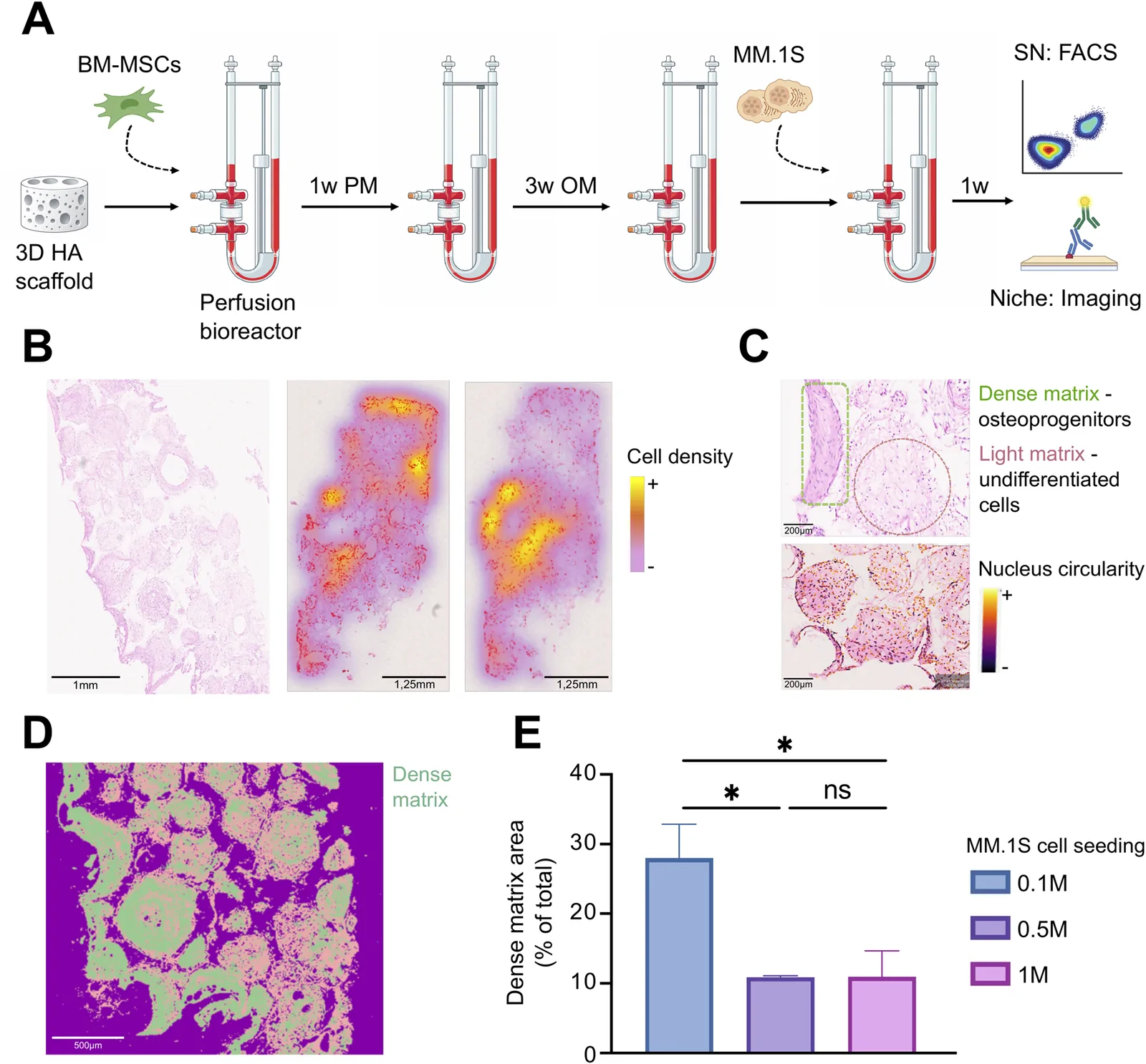
Engineering of a 3D MM model. **(A)** Schematic illustration of the general protocol followed for 3D MM model generation and analysis. **(B)** Representative hematoxylin and eosin (H&E) staining of the 3D MM model at the end of culture period. Cells are marked in red and cell density is visualized as a heatmap. **(C)** Representative H&E staining showing the two different density areas found within scaffold (top) and differences in nucleus circularity within areas (bottom). **(D)** Representative histological section showing dense matrix areas in green. **(E)** Quantification of dense matrix areas among different tumor-seeding conditions. Barplots represent n = 2 biological replicates, with three or more quantification areas each. Quantification areas were averaged for statistical analysis. One-way ANOVA with Tukey’s multiple comparison test **(E)**. ns p.adj>0.05, *p.adj<0.05.

To establish a physiologically relevant 3D MM model, we seeded 3D osteogenic niches previously generated within perfusion bioreactors using BM-MSCs and hydroxyapatite scaffolds (see Methods) with different numbers of MM.1S cells (0.1 million, 0.5 million and one million). As control, MM.1S cells were seeded in cell-free scaffolds. MM.1S cell line was selected because of its capacity to recapitulate the genetic features of primary MM cells and its natural, robust expression of BCMA, which make it an excellent model for assessing CAR T cell therapy targeting this antigen. After 1 week of culture, the perfusion bioreactors were dismounted and samples harvested for end-point analyses. The supernatant fraction was processed for flow cytometry, while the 3D construct (i.e., 3D niches seeded with MM cells) were fixed and processed for imaging analyses (Figure 1A, please see methods for further technical details).

H&E staining at endpoint analysis confirmed that our dynamic cell culture system enabled cellular infiltration and colonization throughout the porous architecture of the hydroxyapatite scaffolds (Figure 1B). We previously showed that, over a 3-week osteogenic differentiation period, BM-MSCs successfully differentiated into osteogenic cells (Bourgine et al., 2018). The histological analysis revealed multiple cells with elongated nuclei and presence of dense extracellular matrix around them, compatible with osteoprogenitors morphology (Figure 1C). Next, we evaluated the impact of MM.1S cells on the stromal osteogenic differentiation by quantifying the density of the deposited matrix (Figure 1D). The area of dense osteogenic-like matrix was superior in presence of the low-tumor-burden condition (0.1 million (0.1M) MM.1S) than in the higher tumor densities (0.5M and 1M MM.1S) (Figure 1E), suggesting a negative correlation between tumor load and osteogenic matrix deposition. These findings suggest that our 3D MM model recapitulates the suppressive effect of MM on osteoblast function, a hallmark of myeloma-related bone disease (Epstein et al., 2002; de Jong et al., 2021; García-Ortiz et al., 2021).

**FIGURE 2:**
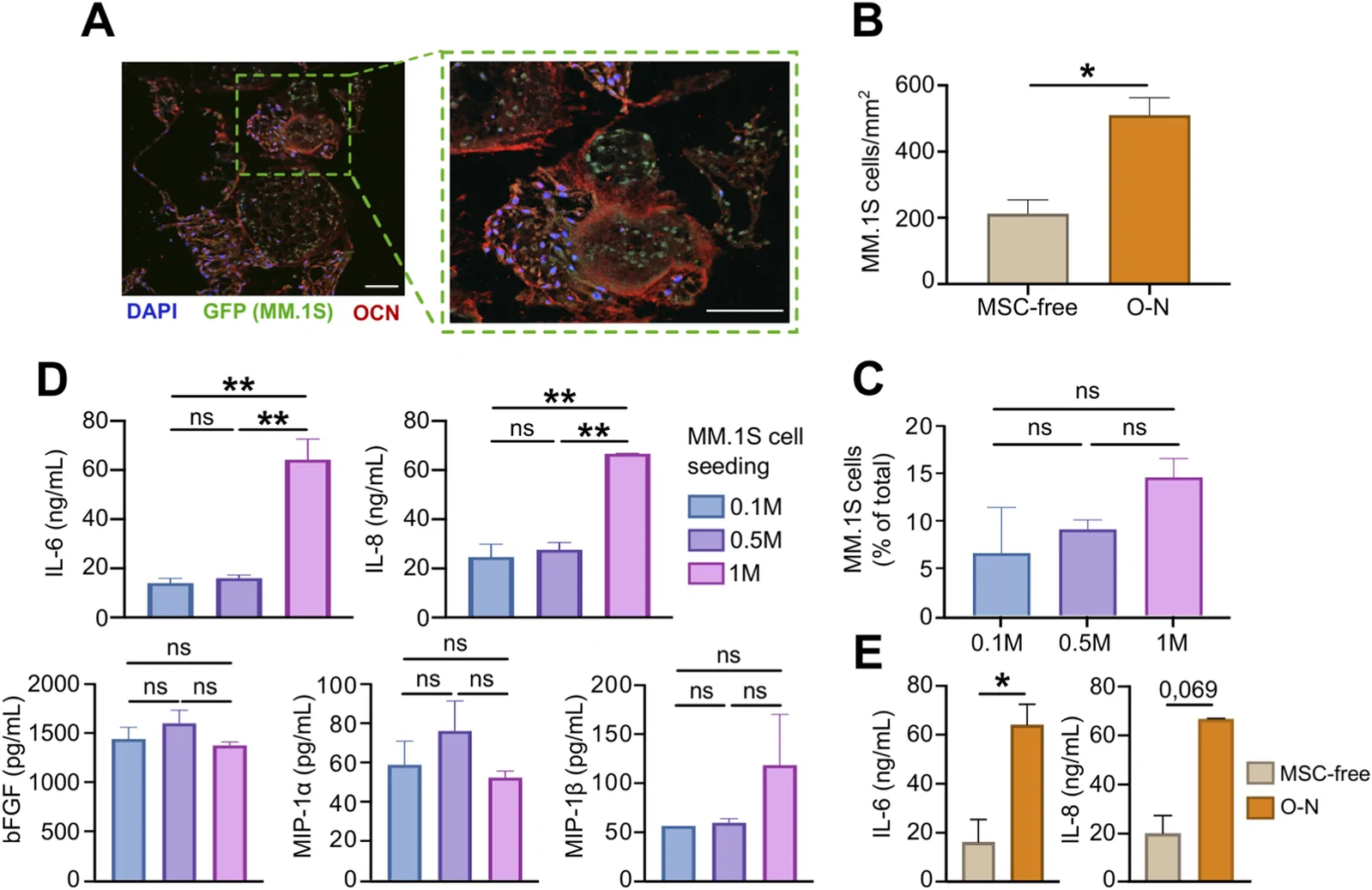
MM cell engraftment within the niche. **(A)** Representative immunofluorescence image of the O-N model after the cell culture period showing tumor cell clusters (GFP, green) within osteocalcin (OCN)-positive regions (red). Nuclei were stained in blue (DAPI). Scale bar, 100 μm. **(B)** Barplot depicting the number of tumor cells identified in histological sections of mesenchymal stromal cell (MSC)-free (n = 2) vs. osteogenic (O-N, n = 2) scaffolds. **(C)** Barplot comparing the percent of tumor cells among different tumor-seeding conditions. Barplots represent n = 2 biological replicates, with three or more quantification areas each. Quantification areas were averaged for statistical analysis. **(D)** Quantification of cytokines in supernatants of 3D MM models at the end of experiment (n = 2 per group). **(E)** Quantification of interleukins in MSC-free and O-N MM niches at the end of experiment (n = 2 per group). Welch’s t-test **(B,E)** and one-way ANOVA with Tukey’s multiple comparisons test **(C,D)**. ns p.adj>0.05, *p.adj<0.05, **p.adj<0.01.

Whole-mount immunofluorescent analysis allowed us to visualize the engraftment of GFP^+^ MM.1S cells within the osteocalcin (OCN)^+^ osteogenic matrix in the 3D MM niche, confirming that the myeloma cells actively homed and engrafted into osteogenic niches. Moreover, the tumor cells self-organized in 3D clusters rather than forming the monolayers typically seen in 2D culture systems (Figure 2A). Engraftment efficiency of MM.1S cells was significantly lower in control MSC-free scaffolds (Figure 2B), pinpointing the crucial role of the osteogenic cells and their extracellular matrix for the correct adherence and survival of MM.1S cells. While the amount of tumor cells initially seeded tended to be proportional to the amount of tumor cells detected later in the MM niche, no significant differences were detected (Figure 2C). Moreover, cytokine analysis in the medium at the end of culture showed increased MM-associated cytokines such as IL-6 and IL-8 in the 3D niches cultured with the highest amount of MM.1S cells, while bFGF, MIP-1α and MIP-1β remained consistent across conditions (Figure 2D). Notably, IL-6 and IL-8 are produced by bone marrow stromal cells in contact with MM cells (Bisping et al., 2003; Herrero et al., 2016); accordingly, we observed increased interleukin levels in bioreactors with O-N niches compared to MSC-free controls (Figure 2E), suggesting MSC-derived production and highlighting the key role of MSCs in supporting the pro-inflammatory MM environment. In summary, our results proved the successful generation of a 3D MM niche that enabled the engraftment of MM cells while recapitulating their suppressive effect on osteogenic cells.

### 3.2 3D MM niches support the maintenance of CAR T compared to UTD cells

After validating the 3D MM model, we aimed to exploit this system for assessing the cytolytic activity of CAR T cells. For this reason, we first assessed the capacity of the 3D MM niches to sustain CAR T cells.

CAR T cells were generated from a healthy donor (see methods). First, to validate the functionality of the BCMA CAR T cell product used in this study, its antitumor efficacy was evaluated in an established MM.1S xenograft model (Supplementary Figure S1B). A single infusion of BCMA CAR T cells induced complete tumor eradication and long-term survival, whereas mice receiving UTD T cells or vehicle rapidly developed progressive disease. Consistent with these *in vivo* results, the manufactured CAR T cells demonstrated superior specific lysis of GFP^+^Luc^+^ MM.1S cells compared with UTD controls in a standard *in vitro* cytotoxicity assay and maintained a predominantly CD4^+^ central memory (TCM) phenotype at the end of production (Supplementary Figure S1C–E).

**FIGURE 3:**
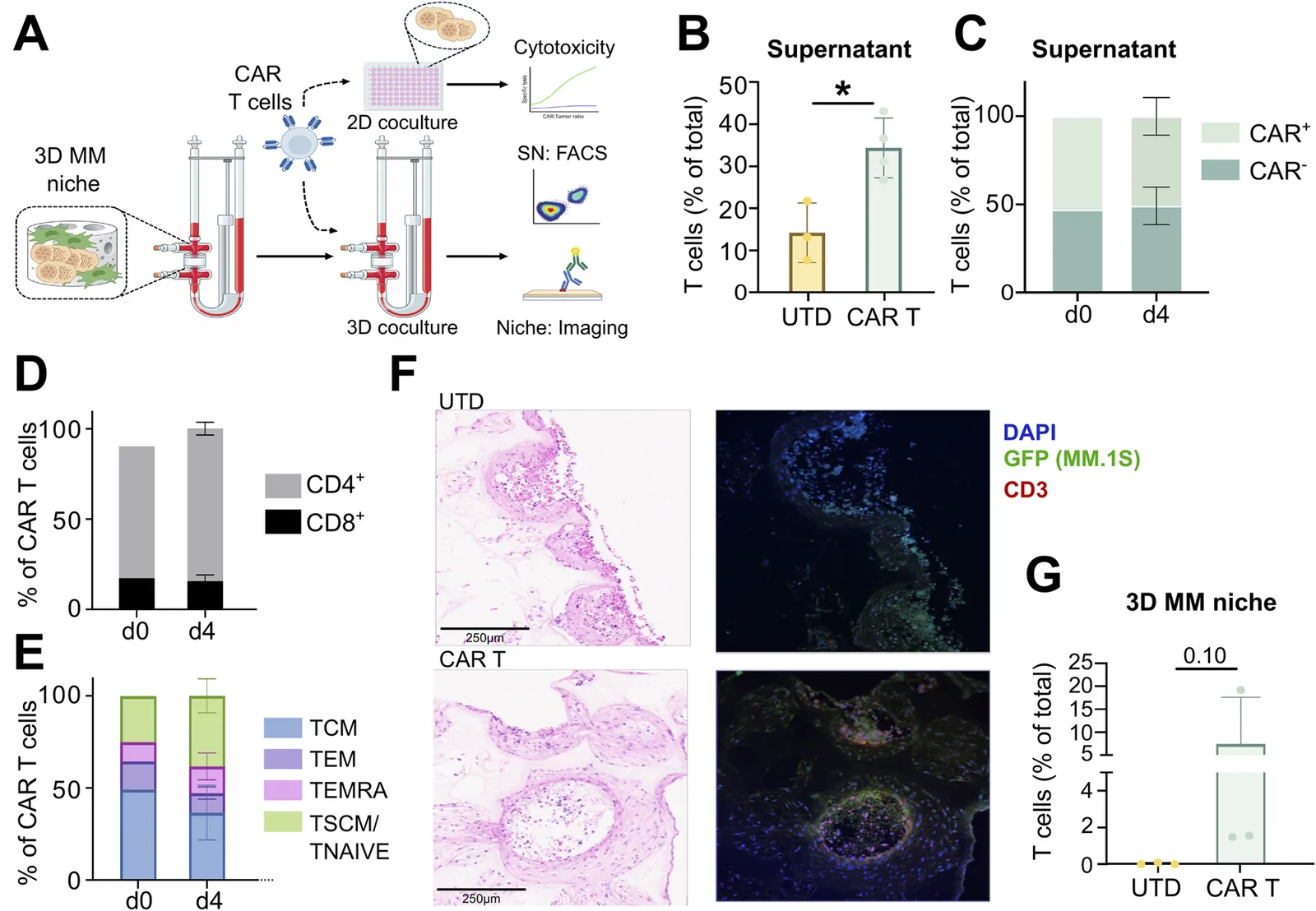
3D MM niches support the maintenance of CAR T cells. **(A)** Schematic illustration of the general protocol followed for CAR T cell coculture within the engineered 3D MM niches. CAR T cell cytotoxicity was simultaneously assessed in a 2D coculture with GFP^+^Luc^+^MM.1S cells. Generated with BioRender. **(B)** Barplot showing the percent of T cells detected by flow cytometry in the supernatants of niches infused with untransduced (UTD) cells (n = 3) or CAR T cells (n = 4). **(C)** Barplot showing the percent of CAR positive and negative (CAR^+^ and CAR^−^, respectively) T cells detected by flow cytometry at the end of production (d0) and in the supernatants 4-day after CAR T cell infusion in the bioreactors (d4). **(D)** Barplots showing membrane expression of CD4^+^/CD8^+^ in CAR T cells at d0 and d4. **(E)** Barplots showing CAR T cell immunophenotype at d0 and d4. (TCM = T central memory, CCR7^+^CD45RA^−^; TEM = T effector memory CCR7^−^CD45RA^−^; TEMRA = Terminal differentiated, CCR7^−^CD45RA^+^; TSCM/TNAIVE = T stem cell memory/T Naïve, CCR7^+^CD45RA^+^). **(F)** Representative hematoxylin and eosin (H&E, left) and immunofluorescence staining (right) of MM niches containing either UTD (top) or CAR (bottom) T cells. Tumor cells are shown in green (GFP), T cells in red (CD3) and nuclei in blue (DAPI). **(G)** Barplot showing the percent of T cells detected within the MM niches in UTD (n = 3) and CAR T (n = 3) groups. Welch’s t-test **(B,C)** and Mann Whitney test **(G)**. *p.adj<0.05, **p.adj<0.005.

Next, CAR T or UTD T cells were infused into bioreactors containing 3D MM niches (Figure 3A). In order to facilitate the interactions between MM cells and CAR T cells in the 3D niche, we set up a 1:1 ratio coculture of MM and CAR T cells (1M MM.1S cells + 1M CAR T cells). After 4 days, the coculture was stopped and we assessed the distribution of CAR T cells both circulating in the fluidic phase of the bioreactor (supernatant) and resident within the tissue (3D MM niche). Flow cytometry analysis of the supernatants showed that the abundance of T cells was significantly higher in those bioreactors seeded with CAR T cells compared to those seeded with UTD controls (Figure 3B). From circulating T cells, around 50% were CAR positive (CAR^+^), maintaining the proportion of the infused product (Figure 3C). Further immunophenotypic analysis of CAR T cells revealed acquisition of a predominant CD4^+^TSCM/TNAIVE phenotype, indicating long-term persistence potential (Xu et al., 2014; Arcangeli et al., 2022; Melenhorst et al., 2022) (Figures 3D,E). Notably, we could only detect CD3^+^ T cells by imaging analysis in the 3D MM niche within those bioreactors seeded with CAR T cells (Figures 3F,G). Collectively, these results suggest that CAR-BCMA interactions facilitate the persistence of CAR T cells in the bioreactor system as well as their recruitment and engraftment in the 3D MM niche.

### 3.3 3D MM niches confer protection to MM.1S cells from CAR T cell killing activity

We then evaluated CAR T cell killing capacity in our MM model. We measured the abundance of MM.1S cells (i) in the supernatant fraction by FACS and (ii) within the 3D MM niche by whole-mount imaging.

**FIGURE 4:**
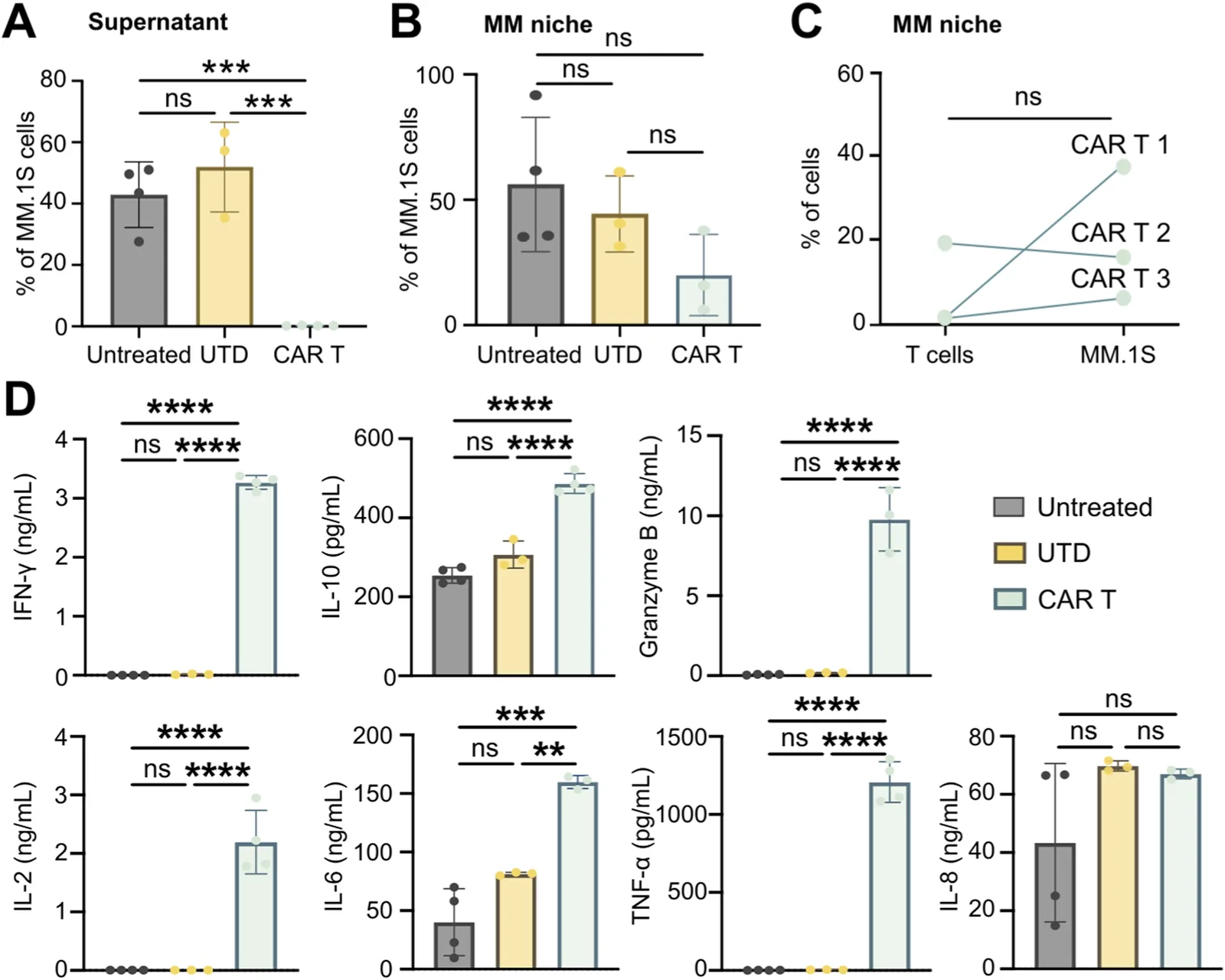
3D MM niches confer protection against CAR T cell killing. Barplot showing percent of MM.1S cells in the supernatant **(A)** or within the scaffold **(B)** of untreated (n = 4), untransduced (UTD) (n = 3) and CAR T cell (n = 3) groups. **(C)** Dotplot showing the proportion of T cells and MM.1S cells within the 3D niche for each individual biological replicate of the CAR T cell condition (n = 3). The paired percentages of T cells and MM.1S cells for each replicate are connected with a line. **(D)** Quantification of cytokines in the supernatant of 3D MM niches at the end of the experiment. One-way ANOVA with Tukey’s multiple comparisons test **(A,B,D)** and paired t-test **(C)**. ns p.adj>0.05, *p.adj<0.05, **p.adj<0.01, ***p.adj<0.001, ****p.adj<0.0001.

In the supernatant fraction, CAR T cells effectively eliminated all circulating MM.1S cells, consistent with both their potent antitumor activity in the xenograft model (Supplementary Figure S1B) and their robust *in vitro* cytotoxicity (Supplementary Figure S1C); and capturing the high killing efficacy that has been described in BCMA CAR T cells (Raje et al., 2019; Sperling et al., 2022; Zhang et al., 2022; Oliver-Caldés et al., 2023). As expected, tumor cells remained intact in the presence of UTD cells (Figure 4A). In contrast, within the 3D MM niche, CAR T cells could only partially clear MM.1S cells, suggesting that the 3D MM niche might elicit a protective effect on MM cells against CAR T cells (Figure 4B). Since the presence of CAR T cells was reduced in the 3D MM niche compared to the supernatant fractions (Figures 3B,G), we further investigated if the decrease in CAR T cell killing activity within the niche could be caused by a reduced T cell recruitment. To this aim, we compared the abundance of T cells and MM.1S cells in each individual sample. The niche hosting the highest proportion of T cells (approx. 20%) was not the one with the lowest tumor burden (Figure 4C), suggesting no direct correlation between the abundance of T cells in the 3D MM niche and CAR T cell killing activity. Since the reduced tumor clearance observed within the engineered 3D MM niche cannot be attributed to limited intrinsic CAR T cell potency, the analysis suggests that MM cells resistance to CAR T cell killing activity is mediated by more complex niche-mediated mechanisms.

Finally, analysis of the supernatants confirmed a drastic release of cytokines related to T cell function in the 3D MM niches seeded with CAR T cells, but not in those untreated or treated with UTD cells. These included high levels of IFNγ, IL-10, Granzyme B, IL-2, IL-6 and TNFα. Interestingly, IL-8 levels did not increase, consistent with the association of IL-8 production with CD28 rather than 4-1BB costimulation (Wechsler et al., 1994; Cook et al., 2025) (Figure 4D).

In summary, our results provided a proof-of-principle for the application of the 3D MM niche as a platform to assess CAR T cell killing efficacy in human settings and recapitulating certain aspects of niche-mediated tumor cell resistance.

## 4 Discussion

In this study we first developed a 3D MM niche model that captures: (i) the engraftment and maintenance of MM cells within an osteogenic matrix, (ii) the suppressive effect of MM cells on osteogenic cells, and (iii) the recruitment and preservation of CAR T cells within the 3D MM niche. Then, we provided initial evidence that the model can be exploited to assess the efficacy of CAR T therapies in a 3D physiological-like environment that provides microenvironmental protection to tumor cells.

We tested a standard experimental setup consisting of culturing MM.1S cells for a week in the 3D osteogenic niche followed by a 4-day therapy period of coculture with CAR T cells. These times might have to be adapted to model specific processes. For example, the culture of MM cells might be extended to further investigate the crosstalk between MM and osteogenic cells, or to model more advanced phases of the pathogenesis. Similarly, longer coculture periods with CAR T cells might enable the investigation of long-term persistence within the niche and potential events of tumor relapse.

To our knowledge, not many studies have reported a 3D MM model integrating osteogenic cells that has been applied to test CAR T cell killing activity. Ghoshal et al. recently reported a multi-niche BM on-a-chip system including both an endosteal (osteogenic) niche and a perivascular niche (Ghoshal et al., 2025). Interestingly, they found that MM cells preferentially localized and proliferated within the perivascular niche compared to the endosteal one, but the mechanisms underlying it remain unknown. Our lab has previously reported a 3D vascularized osteoblastic niche model that could sustain human hematopoiesis (Born et al., 2021). Since osteogenic and vascular cells are physically in contact in this model, the addition of MM cells to this vascularized osteoblastic niche model would enable the further investigation of the interplay between osteogenic, vascular and MM cells by assessing how the vascular component interferes with the crosstalk between MM and osteogenic cells. Moreover, future studies may also add into the system innate immune cells, which are known to impact both MM pathogenesis and response to CAR T cell therapies (Che et al., 2009; Petersson et al., 2021; Yang et al., 2024).

Our results revealed that MM cells negatively impact the osteogenic matrix in the 3D niche, which is consistent with other studies using 3D models that showed that MM cells inhibit mineralization, reduce osteoblastic gene expression and promote osteolysis-related genes (Reagan et al., 2014; Jakubikova et al., 2016; Visconti et al., 2020). Moreover, as previously reported in other studies, our data suggested that MM cells could induce IL-6 and IL-8 production by MSCs (Bisping et al., 2003; Herrero et al., 2016), recapitulating features of the human MM pro-inflammatory environment. Therefore, our data reinforce the concept that MM actively remodels the osteogenic niche and demonstrate that the 3D MM niche model might be applied to dissect the mechanisms underlying this process.

The finding that the 3D MM niche favors the maintenance of CAR T cells over UTD T cells, together with increased T cell presence in the supernatants of CAR T cell samples, suggested that only CAR T cells expanded upon tumor recognition. Further research would be needed to understand whether proliferation preferentially happened within the niche or in the liquid phase, as well as to identify whether interactions with stromal components or cytokine-mediated trafficking promoted CAR T cell infiltration. Since BM-resident CAR T cells have been shown to acquire distinct phenotypic and transcriptomic features compared to circulating counterparts (Jordana-Urriza et al., 2026), it would be of interest to assess whether 3D MM niche-infiltrating CAR T cells recapitulated these features.

The protection of MM cells from CAR T cell killing activity in the 3D niche is aligned with the broader literature showing that the tumor microenvironment confers immune resistance (Kawano et al., 2015). In the specific context of MM, some studies have proposed that stromal cells reduce CAR-immune cell cytotoxicity through paracrine factors and providing a physical barrier (Luanpitpong et al., 2025). Other studies have pointed out the role of cell-to-cell contact and hypoxia in the resistance mechanisms (Damiano et al., 1999; Reagan et al., 2014). The work of Oh and Shen, 2024, for instance, highlights hypoxia as an important biochemical barrier to CAR T function in solid tumors, suggesting that similar mechanisms may also operate in MM (Hu et al., 2012). The design of our bioreactor system allowed us to separately analyze CAR T cell killing activity in the fluidic phase (supernatant) and in the 3D MM niche tissue. Since the protective effect was exclusively detected in the 3D MM niche compartment, our data suggest that cell-adhesion mechanisms and/or physical barrier parameters contribute to tumor resistance, although additional mechanisms such as hypoxia, metabolic competition or immunosuppressive signaling cannot be excluded. Interestingly, despite the reduced tumor clearance observed in the engineered niche 4 days after CAR T cell infusion, the same BCMA CAR T cell product achieved complete tumor eradication in an MM.1S xenograft model. As the *in vivo* study was maintained for 100 days after treatment, these findings likely reflect differences in the temporal evolution of the antitumor response rather than intrinsic CAR T cell potency. Future studies should therefore compare CAR T cell infiltration and tumor burden within the bone marrow at early time points following infusion (e.g., 4 days) to better understand the kinetics of niche-mediated protection *in vivo*. Furthermore, because the xenograft model was established in immunodeficient NSG mice, the contribution of endogenous immune populations to CAR T cell function and immune suppression could not be evaluated. Addressing these mechanisms will require immunocompetent syngeneic MM models, which would enable investigation of the complex interactions between CAR T cells, the tumor microenvironment, and resident immune cells. The model presented here provides a suitable platform for future studies investigating how different microenvironmental factors influence CAR T cell function and contribute to tumor immune evasion.

Despite all these advantages, this is a proof-of-concept study and the presented results should be further validated using larger sample sizes. Moreover, the current model has other limitations. While quantitative flow cytometry analysis was feasible in supernatant fractions, the standard digestion protocol applied to obtain a single cell suspension from the 3D MM niche tissue generated excessive fluorescent debris that challenged both the detection of GFP^+^ MM.1S cells and CAR T cell immunophenotyping. For this reason, we decided to analyze the 3D MM niches exclusively *via* imaging techniques. Further optimization of the digestion protocol and efficient debris removal will be essential to perform reliable FACS and/or transcriptomic analysis in future studies. The co-culture of the 3 cell types (BM-MSCs, MM.1S and CAR T cells) within 3D niches required sequential changes in media composition to fulfill their different nutritional requirements. Future studies might systematically evaluate the impact of the different media compositions on each cell type separately. Additionally, in this study the BM-MSCs used to generate the 3D MM niche and the T cells later engineered to generate CAR T cells were derived from different donors. Although BM-MSCs and derived cell lineages are well-known to exhibit immunomodulatory properties (Shi et al., 2011), HLA compatibility was not assessed between the different donor cells, and therefore we cannot exclude the contribution of some graft-versus-host reaction in our system. Even if we did not observe any apparent detrimental effect on the 3D osteogenic niche upon T cell infusion into the system, this issue could be circumvented by isolating the BM-MSCs and T cells from the same donor, or by performing a TCR depletion of CAR T cells (Mailankody et al., 2023). The generation of the 3D MM model and CAR T cells from a single donor would enable to generate a personalized platform for patient-specific testing of CAR T therapies. Further, future studies may evaluate the ability of this platform to support patient-derived MM cells and autologous CAR T cells, enabling personalized assessment of tumor–microenvironment interactions and therapeutic responses. Finally, the implementation of bioprinting techniques might improve the throughput capacity of the platform and enhance its predictive power for immunotherapy (Wu et al., 2022).

## 5 Conclusion

This proof-of-concept 3D MM model positions at the intersection of two unmet needs in the context of MM: (i) modeling the disease in a complex, fully-human 3D system capturing the dynamic interactions with the osteogenic microenvironment, and (ii) providing a platform to assess CAR T cell therapy efficacy in presence of niche-mediated tumor resistance. Its application to evaluate next-generation CAR designs and microenvironment-targeted agents might enable to address biological mechanistic questions and to more accurately predict patient outcomes.

## Data Availability

The original contributions presented in the study are included in the article/Supplementary Material, further inquiries can be directed to the corresponding author.

## Supplementary material

SUPPLEMENTARY FIGURE S1 CAR T cell characterization. **(A)** Schematic illustration of the general protocol followed for CAR T cell production. **(B)** Kaplan-Meier survival curve of NSG mice intravenously inoculated with 3×10^6^ MM.1S cells on day 0 and treated on day 14 with 0.5×10^6^ BCMA CAR T cells (n = 6), untransduced (UTD) T cells (n = 4) or left untreated (n = 4). Statistical significance was determined using the log-rank (Mantel–Cox) test. **(C)** Specific lysis of CAR T and UTD cells after 24h coculture with GFPLuc+MM.1S cells at different effector:target (E:T) ratios. **(D)** Barplot showing membrane CD4+/CD8+ expression in T cells at basal state (before CAR T cell production) and at the end of CAR T cell production (d0). **(E)** Barplot showing T cell immunophenotypes at basal state and at d0 (TCM = T central memory, CCR7+CD45RA-; TEM = T effector memory CCR7-CD45RA-; TEMRA = Terminal differentiated, CCR7-CD45RA+ ; TSCM/TNAIVE = T stem cell memory / T Naïve, CCR7+CD45RA+). ***p<0.001.

## References

[B1] Arcangeli S. Bove C. Mezzanotte C. Camisa B. Falcone L. Manfredi F. (2022). CAR T cell manufacturing from naive/stem memory T lymphocytes enhances antitumor responses while curtailing cytokine release syndrome. J. Clin. Investigation 132, e150807. 10.1172/JCI150807 35503659 PMC9197529

[B2] Arnulf B. Lecourt S. Soulier J. Ternaux B. Lacassagne M. N. Crinquette A. (2007). Phenotypic and functional characterization of bone marrow mesenchymal stem cells derived from patients with multiple myeloma. Leukemia 21, 158–163. 10.1038/sj.leu.2404466 17096013

[B3] Berdeja J. G. Madduri D. Usmani S. Z. Jakubowiak A. Agha M. Cohen A. D. (2021). Ciltacabtagene autoleucel, a B-cell maturation antigen-directed chimeric antigen receptor T-cell therapy in patients with relapsed or refractory multiple myeloma (CARTITUDE-1): a phase 1b/2 open-label study. Lancet 398, 314–324. 10.1016/S0140-6736(21)00933-8 34175021

[B4] Bisping G. Leo R. Wenning D. Dankbar B. Padró T. Kropff M. (2003). Paracrine interactions of basic fibroblast growth factor and interleukin-6 in multiple myeloma. Blood 101, 2775–2783. 10.1182/blood 12517814

[B5] Born G. Nikolova M. Scherberich A. Treutlein B. García-García A. Martin I. (2021). Engineering of fully humanized and vascularized 3D bone marrow niches sustaining undifferentiated human cord blood hematopoietic stem and progenitor cells. J. Tissue Eng. 12, 20417314211044855. 10.1177/20417314211044855 34616539 PMC8488506

[B6] Boulch M. Cazaux M. Loe-Mie Y. Thibaut R. Corre B. Lemaître F. (2021). A crosstalk between CAR T cell subsets and the tumor microenvironment is essential for sustained cytotoxic activity. Sci. Immunol. 6, eabd4344. 10.1126/sciimmunol.abd4344 33771887

[B7] Bourgine P. E. Klein T. Paczulla A. M. Shimizu T. Kunz L. Kokkaliaris K. D. (2018). *In vitro* biomimetic engineering of a human hematopoietic niche with functional properties. Proc. Natl. Acad. Sci. U. S. A. 115, E5688–E5695. 10.1073/pnas.1805440115 29866839 PMC6016789

[B8] Braham M. V. J. Ahlfeld T. Akkineni A. R. Minnema M. C. Dhert W. J. A. Öner F. C. (2018). Endosteal and perivascular subniches in a 3D bone marrow model for multiple myeloma. Tissue Eng. Part C Methods 24, 300–312. 10.1089/ten.tec.2017.0467 29652626

[B9] Che H. Campbell R. A. Chang Y. Li M. Wang C. S. Li J. (2009). Pleiotrophin produced by multiple myeloma induces transdifferentiation of monocytes into vascular endothelial cells: a novel mechanism of tumor-induced vasculogenesis. Blood 113, 1992–2002. 10.1182/blood-2008-02-133751 19060246 PMC2651013

[B10] Cook M. S. King E. Flaherty K. R. Siddika K. Papa S. Benjamin R. (2025). CAR-T cells containing CD28 *versus* 4-1BB co-stimulatory domains show distinct metabolic profiles in patients. Cell Rep. 44, 115973. 10.1016/j.celrep.2025.115973 40650909

[B11] Corre J. Mahtouk K. Attal M. Gadelorge M. Huynh A. Fleury-Cappellesso S. (2007). Bone marrow mesenchymal stem cells are abnormal in multiple myeloma. Leukemia 21, 1079–1088. 10.1038/sj.leu.2404621 17344918 PMC2346535

[B12] Da Vià M. C. Dietrich O. Truger M. Arampatzi P. Duell J. Heidemeier A. (2021). Homozygous BCMA gene deletion in response to anti-BCMA CAR T cells in a patient with multiple myeloma. Nat. Med. 27, 616–619. 10.1038/s41591-021-01245-5 33619368

[B13] Damiano J. S. Cress A. E. Hazlehurst L. A. Shtil A. A. Dalton W. S. (1999). Cell adhesion mediated drug resistance (CAM-DR): role of integrins and resistance to apoptosis in human myeloma cell lines. Blood 93, 1658–1667. 10.1182/blood.V93.5.1658 10029595 PMC5550098

[B14] de Jong M. M. E. Kellermayer Z. Papazian N. Tahri S. Hofste op Bruinink D. Hoogenboezem R. (2021). The multiple myeloma microenvironment is defined by an inflammatory stromal cell landscape. Nat. Immunol. 22, 769–780. 10.1038/s41590-021-00931-3 34017122

[B15] de la Puente P. Muz B. Gilson R. C. Azab F. Luderer M. King J. (2015). 3D tissue-engineered bone marrow as a novel model to study pathophysiology and drug resistance in multiple myeloma. Biomaterials 73, 70–84. 10.1016/j.biomaterials.2015.09.017 26402156 PMC4917006

[B16] Epstein J. Barlogie B. Johnson C. L. Yaccoby S. Pearse R. N. Choi Y. (2002). Myeloma interacts with the bone marrow microenvironment to induce osteoclastogenesis and is dependent on osteoclast activity. Br. J. Haematol. 116, 278–290. 10.1046/j.1365-2141.2002.03257.x 11841428

[B17] Garayoa M. Garcia J. L. Santamaria C. Garcia-Gomez A. Blanco J. F. Pandiella A. (2009). Mesenchymal stem cells from multiple myeloma patients display distinct genomic profile as compared with those from normal donors. Leukemia 23, 1515–1527. 10.1038/leu.2009.65 19357701

[B18] García-Ortiz A. Rodríguez-García Y. Encinas J. Maroto-Martín E. Castellano E. Teixidó J. (2021). The role of tumor microenvironment in multiple Myeloma development and progression. Cancers (Basel) 13, 217. 10.3390/cancers 33435306 PMC7827690

[B19] Garfall A. L. Dancy E. K. Cohen A. D. Hwang W. T. Fraietta J. A. Davis M. M. (2019). T-cell phenotypes associated with effective CAR T-cell therapy in postinduction vs relapsed multiple myeloma. Blood Adv. 3, 2812–2815. 10.1182/bloodadvances.2019000600 31575532 PMC6784521

[B20] Ghoshal D. Petersen I. Ringquist R. Kramer L. Bhatia E. Hu T. (2025). Multi-niche human bone marrow on-a-chip for studying the interactions of adoptive CAR-T cell therapies with multiple myeloma. Biomaterials 316, 123016. 10.1016/j.biomaterials.2024.123016 39709851

[B21] Herrero A. B. García-Gómez A. Garayoa M. Corchete L. A. Hernández J. M. San Miguel J. (2016). Effects of IL-8 Up-Regulation on cell survival and osteoclastogenesis in multiple myeloma. Am. J. Pathology 186, 2171–2182. 10.1016/j.ajpath.2016.04.003 27301357

[B22] Hu J. Van Valckenborgh E. Menu E. De Bruyne E. Vanderkerken K. (2012). Understanding the hypoxic niche of multiple myeloma: therapeutic implications and contributions of mouse models. DMM Dis. Models Mech. 5, 763–771. 10.1242/dmm.008961 23115205 PMC3484859

[B23] Ilic J. Koelbl C. Simon F. Wußmann M. Ebert R. Trivanovic D. (2024). Liquid overlay and collagen-based three-dimensional models for *in vitro* investigation of multiple myeloma. Tissue Eng. Part C Methods 30, 193–205. 10.1089/ten.tec.2023.0374 38545771

[B24] Jakubikova J. Cholujova D. Hideshima T. Gronesova P. Soltysova A. Harada T. (2016). A novel 3D mesenchymal stem cell model of the multiple myeloma bone marrow niche: biologic and clinical applications. Oncotarget 7, 77326–77341. 10.18632/oncotarget.12643 27764795 PMC5357212

[B25] Jordana-Urriza L. Serrano G. Camara-Peña S. Calleja-Cervantes M. E. San Martin-Uriz P. Zabaleta A. (2026). Single-Cell multiomics reveals regulatory mechanisms of CAR T cell persistence and dysfunction in multiple myeloma. Blood Neoplasia 3, 100203. 10.1016/j.bneo.2026.100203 42027747 PMC13101692

[B26] Karadag O. Apperley R. (2000). Human myeloma cells promote the production of interleukin 6 by primary human osteoblasts. Br. J. Haematol. 108, 383–390. 10.1046/j.1365-2141.2000.01845.x 10691869

[B27] Kawano Y. Moschetta M. Manier S. Glavey S. Gö G. T. Roccaro A. M. (2015). Targeting the bone marrow microenvironment in multiple myeloma. Immunol. Rev. 263, 160–172. 10.1111/imr.12233 25510276

[B28] Lee H. Ahn S. Maity R. Leblay N. Ziccheddu B. Truger M. (2023). Mechanisms of antigen escape from BCMA- or GPRC5D-targeted immunotherapies in multiple myeloma. Nat. Med. 29, 2295–2306. 10.1038/s41591-023-02491-5 37653344 PMC10504087

[B29] Li M. Xue S. L. Tang X. Xu J. Chen S. Han Y. (2022). The differential effects of tumor burdens on predicting the net benefits of ssCART-19 cell treatment on r/r B-ALL patients. Sci. Rep. 12, 378. 10.1038/s41598-021-04296-3 35013456 PMC8748521

[B30] Lourenço D. Lopes R. Pestana C. Queirós A. C. João C. Carneiro E. A. (2022). Patient-Derived multiple myeloma 3D models for personalized Medicine—Are we there yet? Int. J. Mol. Sci. 23, 12888. 10.3390/ijms232112888 36361677 PMC9657251

[B31] Luanpitpong S. Janan M. Poohadsuan J. Rodboon N. Samart P. Rungarunlert S. (2025). A High-Throughput, three-dimensional multiple myeloma model recapitulating tumor-stroma interactions for CAR-Immune cell-mediated cytotoxicity assay. Immunotargets Ther. 14, 321–338. 10.2147/ITT.S503984 40182067 PMC11967349

[B32] Mailankody S. Matous J. V. Chhabra S. Liedtke M. Sidana S. Oluwole O. O. (2023). Allogeneic BCMA-targeting CAR T cells in relapsed/refractory multiple myeloma: phase 1 UNIVERSAL trial interim results. Nat. Med. 29, 422–429. 10.1038/s41591-022-02182-7 36690811

[B33] Malard F. Neri P. Bahlis N. J. Terpos E. Moukalled N. Hungria V. T. M. (2024). Multiple myeloma. Nat. Rev. Dis. Prim. 10, 45. 10.1038/s41572-024-00529-7 38937492

[B34] Melenhorst J. J. Chen G. M. Wang M. Porter D. L. Gao P. Bandyopadhyay S. (2022). Decade-long leukemia remissions with persistence of CD4+ CAR T cells. Nature 602, 503–509. 10.1101/2021.05.07.443194 35110735 PMC9166916

[B35] Mirvis E. Benjamin R. (2024). Are we there yet? CAR-T therapy in multiple myeloma. Br. J. Haematol. 205, 2175–2189. 10.1111/bjh.19896 39558776 PMC11637742

[B36] Monfrini C. Stella F. Aragona V. Magni M. Ljevar S. Vella C. (2022). Phenotypic composition of commercial Anti-CD19 CAR T cells affects *in vivo* expansion and disease response in patients with large B-cell lymphoma. Clin. Cancer Res. 28, 3378–3386. 10.1158/1078-0432.CCR-22-0164 35583610 PMC9662896

[B37] Munshi N. C. Anderson L. D. Shah N. Madduri D. Berdeja J. Lonial S. (2021). Idecabtagene vicleucel in relapsed and refractory multiple myeloma. N. Engl. J. Med. 384, 705–716. 10.1056/nejmoa2024850 33626253

[B38] Oh J. M. Shen K. (2024). Hypoxic 3D tumor model for evaluating of CAR-T cell therapy *in vitro* . Methods Mol. Biol. 2748, 119–134. 10.1007/978-1-0716-3593-3_10 38070112 PMC12790269

[B39] Oliver-Caldés A. González-Calle V. Cabañas V. Español-Rego M. Rodríguez-Otero P. Reguera J. L. (2023). Fractionated initial infusion and booster dose of ARI0002h, a humanised, BCMA-directed CAR T-cell therapy, for patients with relapsed or refractory multiple myeloma (CARTBCMA-HCB-01): a single-arm, multicentre, academic pilot study. Lancet Oncol. 24, 913–924. 10.1016/S1470-2045(23)00222-X 37414060

[B40] Petersson J. Askman S. Pettersson Å. Wichert S. Hellmark T. Johansson Å. C. M. (2021). Bone marrow neutrophils of multiple myeloma patients exhibit myeloid-derived suppressor cell activity. J. Immunol. Res. 2021, 6344344. 10.1155/2021/6344344 34414242 PMC8369183

[B41] Raje N. Berdeja J. Lin Y. Siegel D. Jagannath S. Madduri D. (2019). Anti-BCMA CAR T-Cell therapy bb2121 in relapsed or refractory multiple myeloma. N. Engl. J. Med. 380, 1726–1737. 10.1056/nejmoa1817226 31042825 PMC8202968

[B42] Reagan M. R. Mishima Y. Glavey S. V. Zhang Y. Manier S. Lu Z. N. (2014). Investigating osteogenic differentiation in multiple myeloma using a novel 3D bone marrow niche model. Blood 124, 3250–3259. 10.1182/blood-2014-02-558007 25205118 PMC4239334

[B43] Rodriguez-Otero P. Ailawadhi S. Arnulf B. Patel K. Cavo M. Nooka A. K. (2023). Ide-cel or standard regimens in relapsed and refractory multiple myeloma. N. Engl. J. Med. 388, 1002–1014. 10.1056/nejmoa2213614 36762851

[B44] Samur M. K. Fulciniti M. Aktas Samur A. Bazarbachi A. H. Tai Y. T. Prabhala R. (2021). Biallelic loss of BCMA as a resistance mechanism to CAR T cell therapy in a patient with multiple myeloma. Nat. Commun. 12, 868. 10.1038/s41467-021-21177-5 33558511 PMC7870932

[B45] San-Miguel J. Dhakal B. Yong K. Spencer A. Anguille S. Mateos M.-V. (2023). Cilta-cel or standard care in Lenalidomide-Refractory multiple myeloma. N. Engl. J. Med. 389, 335–347. 10.1056/nejmoa2303379 37272512

[B46] Shah N. N. Fry T. J. (2019). Mechanisms of resistance to CAR T cell therapy. Nat. Rev. Clin. Oncol. 16, 372–385. 10.1038/s41571-019-0184-6 30837712 PMC8214555

[B47] Shi M. Liu Z.-W. Wang F.-S. (2011). Immunomodulatory properties and therapeutic application of mesenchymal stem cells. Clin. Exp. Immunol. 164, 1–8. 10.1111/j.1365-2249.2011.04327.x 21352202 PMC3074211

[B48] Sperling A. S. Nikiforow S. Derman B. Nadeem O. Mo C. Laubach J. (2022). “P1446 phase I study data update of PHE885, a fully human BCMA-directed CAR-T cell therapy manufactured using the T-ChargeTM platform for patients with relapsed/refractory (R/R) Multiple Myeloma (MM),” in *Gene Therapy, Cellular Immunotherapy and Vaccination*, (Hemasphere). Available online at: https://journals.lww.com/hemasphere/pages/default.aspx (Accessed April, 2026).

[B49] Turtle C. J. Hanafi L. A. Berger C. Hudecek M. Pender B. Robinson E. (2016). Immunotherapy of non-hodgkin’s lymphoma with a defined ratio of CD8+ and CD4+ CD19-specific chimeric antigen receptor-modified T cells. Sci. Transl. Med. 8, 355ra116. 10.1126/scitranslmed.aaf8621 27605551 PMC5045301

[B50] Visconti R. J. Kolaja K. Cottrell J. A. (2020). A functional three-dimensional microphysiological human model of myeloma bone disease. J. Bone Mineral Res. 36, 1914–1930. 10.1002/jbmr.4404 34173283

[B51] Wang J. Hendrix A. Hernot S. Lemaire M. De Bruyne E. Valckenborgh E. V. (2014). Bone marrow stromal cell-derived exosomes as communicators in drug resistance in multiple myeloma cells. Blood 124, 555–566. 10.1182/blood-2014-03-562439 24928860

[B52] Wechsler A. S. Gordon M. C. Dendorfer U. LeClair K. P. (1994). Induction of IL-8 expression in T cells uses the CD28 costimulatory pathway. J. Immunol. 153, 2515–2523. 10.4049/jimmunol.153.6.2515 8077662

[B53] Wu D. Wang Z. Li J. Song Y. Perez M. E. M. Wang Z. (2022). A 3D-Bioprinted multiple myeloma model. Adv. Healthc. Mater. 11, e2100884. 10.1002/adhm.202100884 34558232 PMC8940744

[B54] Xu Y. Zhang M. Ramos C. A. Durett A. Liu E. Dakhova O. (2014). Closely related T-memory stem cells correlate with *in vivo* expansion of CAR.CD19-T cells and are preserved by IL-7 and IL-15. Blood 123, 3750–3759. 10.1182/blood-2014-01-552174 24782509 PMC4055922

[B55] Yang S. Xu J. Dai Y. Jin S. Sun Y. Li J. (2024). Neutrophil activation and clonal CAR-T re-expansion underpinning cytokine release syndrome during ciltacabtagene autoleucel therapy in multiple myeloma. Nat. Commun. 15, 360. 10.1038/s41467-023-44648-3 38191582 PMC10774397

[B56] Zhang W. Lee W. Y. Siegel D. S. Tolias P. Zilberberg J. (2014). Patient-Specific 3D microfluidic tissue model for multiple myeloma. Tissue Eng. Part C Methods 20, 663–670. 10.1089/ten.tec.2013.0490 24294886

[B57] Zhang X. Zhu L. Zhang H. Chen S. Xiao Y. (2022). CAR-T cell therapy in hematological malignancies: current opportunities and challenges. Front. Immunol. 13, 1–20. 10.3389/fimmu.2022.927153 35757715 PMC9226391

[B58] Zheng Y. Chow S.-O. Boernert K. Basel D. Mikuscheva A. Kim S. (2014). Direct crosstalk between cancer and osteoblast lineage cells fuels metastatic growth in bone *via* auto-amplification of IL-6 and RANKL signaling pathways. J. Bone Mineral Res. 29, 1938–1949. 10.1002/jbmr.2231 24676805

